# Women, children and adolescents in conflict countries: an assessment of inequalities in intervention coverage and survival

**DOI:** 10.1136/bmjgh-2019-002214

**Published:** 2020-01-26

**Authors:** Nadia Akseer, James Wright, Hana Tasic, Karl Everett, Elaine Scudder, Ribka Amsalu, Ties Boerma, Eran Bendavid, Mahdis Kamali, Aluisio J D Barros, Inácio Crochemore Mohnsam da Silva, Zulfiqar Ahmed Bhutta

**Affiliations:** 1 Centre for Global Child Health, Hospital for Sick Children, Toronto, Ontario, Canada; 2 Save the Children USA, Washington, District of Columbia, USA; 3 Rady Faculty of Health Sciences, University of Manitoba, Winnipeg, MB, Canada; 4 Centers for Health Policy, Primary Care and Outcomes Research, Stanford University, Stanford, California, USA; 5 International Centre for Equity in Health, Postgraduate Program in Epidemiology, Federal University of Pelotas, Pelotas, Brazil

**Keywords:** maternal health, child health

## Abstract

**Introduction:**

Conflict adversely impacts health and health systems, yet its effect on health inequalities, particularly for women and children, has not been systematically studied. We examined wealth, education and urban/rural residence inequalities for child mortality and essential reproductive, maternal, newborn and child health interventions between conflict and non-conflict low-income and middle-income countries (LMICs).

**Methods:**

We carried out a time-series multicountry ecological study using data for 137 LMICs between 1990 and 2017, as defined by the 2019 World Bank classification. The data set covers approximately 3.8 million surveyed mothers (15–49 years) and 1.1 million children under 5 years including newborns (<1 month), young children (1–59 months) and school-aged children and adolescents (5–14 years). Outcomes include annual maternal and child mortality rates and coverage (%) of family planning services, 1+antenatal care visit, skilled attendant at birth (SBA), exclusive breast feeding (0–5 months), early initiation of breast feeding (within 1 hour), neonatal protection against tetanus, newborn postnatal care within 2 days, 3 doses of diphtheria, pertussis and tetanus vaccine, measles vaccination, and careseeking for pneumonia and diarrhoea.

**Results:**

Conflict countries had consistently higher maternal and child mortality rates than non-conflict countries since 1990 and these gaps persist despite rates continually declining for both groups. Access to essential reproductive and maternal health services for poorer, less educated and rural-based families was several folds worse in conflict versus non-conflict countries.

**Conclusions:**

Inequalities in coverage of reproductive**/**maternal health and child vaccine interventions are significantly worse in conflict-affected countries. Efforts to protect maternal and child health interventions in conflict settings should target the most disadvantaged families including the poorest, least educated and those living in rural areas.

**Key messages**

**What is already known?**

- Maternal, newborn, child and adolescent death rates are higher in chronic conflict countries.
- The poorest and rural households suffer higher child mortality rates in conflict countries compared with their counterparts in non-conflict countries.
- Conflict countries have notably lower coverage of reproductive and maternal health services and childhood vaccinations as compared with non-conflict countries.

**What are the new findings?**

- Inequalities in both conflict and non-conflict countries tend to favour the rich, most educated and urban populations to varying degrees.
- Inequalities with respect to wealth quintile, maternal education level and urban/rural populations are significantly higher in conflict countries for interventions including family planning, antenatal care, skilled birth attendance and childhood vaccination as compared with non-conflict countries.
- Inequalities by wealth, maternal education and residence in postnatal newborn care, breastfeeding practices and utilisation of curative child interventions (for pneumonia and diarrhoea) are minor and differ marginally between conflict and non-conflict countries.

**What do the new findings imply?**

- Donors, NGOs, policy makers and civil society in fragile contexts should prioritise efforts to reduce health inequities including implementing community-based outreach initiatives, harnessing state and non-state resources to provide health services, adopting innovative modalities for scaling health intervention at risk of inequality, tackling emerging health priorities and developing pro-equity health policies.

## Introduction

**Figure 1:**
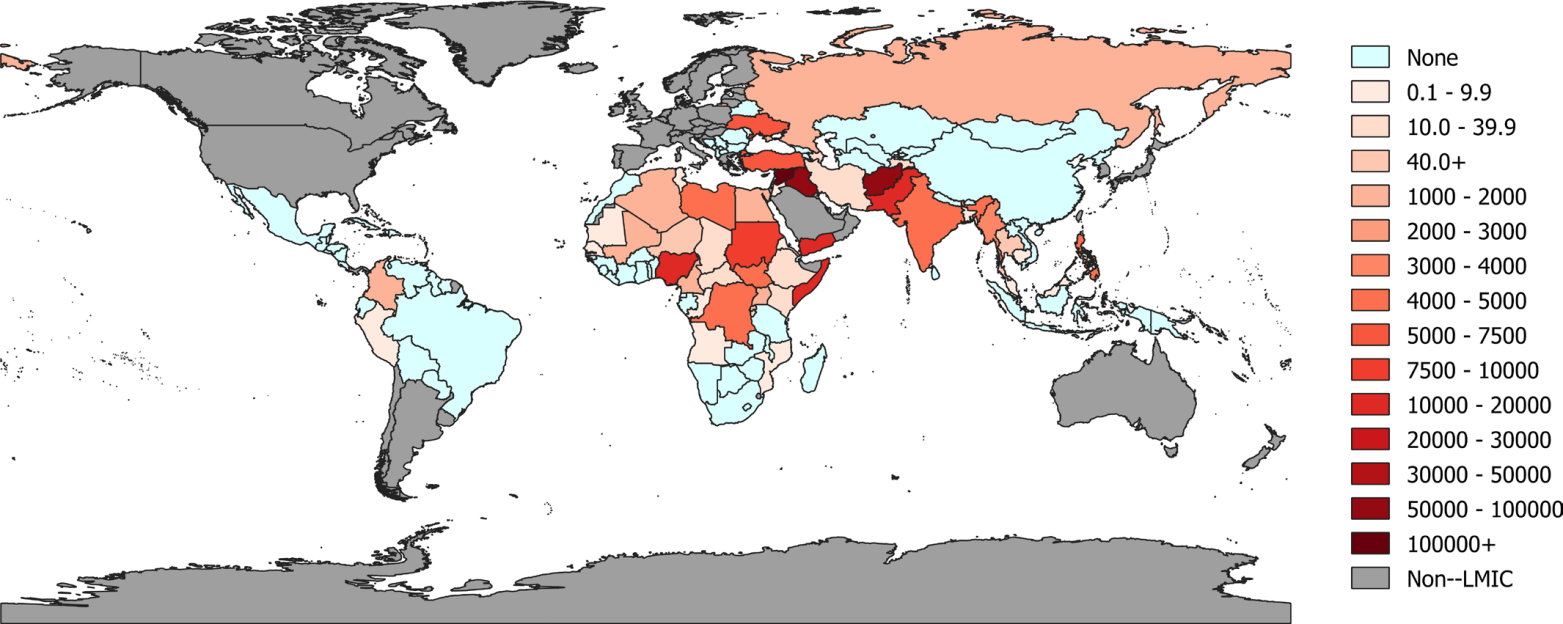
Numbers of battle-related deaths in low-income and middle-income countries (2010–2017) (adapted from Uppsala Conflict Data Program 2019 [5]).

The effects of war and conflict are profound and far-reaching, extending well beyond the battlefield, country borders and period of active warfare. In addition to claiming many lives, conflict has the ability to shatter political, economic and social institutions and adversely affects a nation’s opportunities for sustainable development.^1^ The nature of conflict has changed dramatically over the years. Today, most armed conflicts occur *within* countries rather than *between* countries and often civilian victims, mostly women and children, outnumber combatant casualties.^2 3^ Recent estimates have also shown that three quarters of those in need of humanitarian assistance are women and children.^4^ Deaths directly due to war and conflict—often referred to as battle-related deaths (BRD)—remain high, and are particularly clustered in low-income and middle-income countries (LMICs) of Africa, the Middle East and South Asia (figure 1), such as Syria (270 553 deaths between 2010 and 2017), Afghanistan (96 654), Iraq (55 236), Pakistan (19 253), Yemen (17 539) and Nigeria (16 475).^5^

That pervasive conflict and violence adversely affect the health and well-being of populations, both directly and indirectly, is well recognised.^6^ Deteriorating structural and social determinants of health, including economic, social, cultural, environmental and political systems, are commonplace in such settings; this may hinder the provision of healthcare and public resources to the poorest and exacerbate health disparities.^7 8^

However, the extent to which women and children experience disproportionate death and health inequalities in conflict countries has not been systematically studied. Using existing literature and empirical analyses, this paper addresses this gap.

We conducted a systematic study of the levels and trends in maternal, child and adolescent survival and health service inequalities in conflict versus non-conflict LMICs using an ecological (multinational) approach from 1990 to 2017. We begin by contrasting the mortality of mothers and children in conflict countries relative to non-conflict countries. Among civilian populations, often the most disadvantaged mothers and children, especially the least educated,^9 10^ the poorest^9–12^ and those living in remote/inaccessible areas,^9 10 12^ are at highest risk of adversity in conflict settings. We focus on these dimensions of inequality in this study. Next, the dimensions of health inequality (eg, wealth, education, rurality) that are at particular risk for exacerbation in conflict zones and their underlying mechanisms are discussed. Subsequently, we present systematic comparisons of coverage inequalities for essential reproductive, maternal, newborn and child health interventions between conflict and non-conflict countries, with support from published literature. We close with a discussion of priorities and practical solutions for targeting health inequities in fragile contexts. It should be noted that our study aimed to assess the absolute burden of differences between conflict and non-conflict countries; we do not examine causality nor underlying pathways to the differences.

## Methods

### Study design

We conducted a time-series multicountry ecological study of associations between conflict severity and maternal and child health outcomes in all LMICs as defined by the 2019 World Bank classification.

### Conflict country classification

BRD data from the Uppsala Conflict Data Program (UCDP)^5^ were used to classify conflict status of each country. Death count data were converted into a battle-related mortality rate per million individuals per year. For neonatal, under 5 years and maternal mortality rate trends, annual classifications of ‘Conflict’ vs ‘Non-conflict’ were derived for all LMICs for each year 1990–2017, where ‘Conflict’ was defined as having a battle-related mortality rate of at least 10 per million and ‘Non-conflict’ otherwise. Due to scarcity of intervention coverage data, the impact of sustained periods of conflict on health intervention inequality was assessed with slightly different conflict status definitions. Here, we defined a ‘Conflict’ country as one with a mean battle-related mortality rate of at least 10 per million for each decade between 1990–2017 (ie, 1990–1999, 2000–2009, 2010–2017), else ‘Non-conflict’. See illustrative calculations and final country conflict ranking in the online supplementary tables A1-A4. The sensitivity of this cut-off value was evaluated in a consultative process using both quantitative and qualitative insights, such as UCDP data^5^ and the World Bank’s Harmonised List of Fragile States.^13^

10.1136/bmjgh-2019-002214.supp1 Supplementary data

### Outcomes

We studied mortality of mothers (15–49 years), newborns (<1 month), young children (1–59 months), and school-aged children and adolescents (5–14 years). Annual mortality rates were plotted against time for all countries, stratified by conflict status, and a local polynomial smoothing function calculated the best fit for trend over time. We also examined inequalities in essential maternal, newborn and child health interventions including family planning (defined as demand satisfied with modern methods), at least one+antenatal care visit (ANC), skilled attendant at birth (SBA), exclusive breastfeeding (0–5 months), early initiation of breastfeeding (within 1 hour), neonatal protection against tetanus, newborn postnatal care within 2 days, 3 doses of diphtheria, pertussis and tetanus vaccine (DPT3), measles vaccination, and careseeking for pneumonia and diarrhoea.

### Literature review

We searched PubMed, Medline and Scopus databases for literature on reproductive, maternal, infant, child and adolescent health inequality in conflict settings. MeSH terms included (‘war’ OR ‘conflict’) AND (‘women’s health’ OR ‘child health’ OR ‘infant health’ OR ‘adolescent health’) AND (‘inequality’ OR ‘socioeconomic factor’). The search was conducted in June 2019 and was not restricted by year or language.

### Data sources and variables

Several online databases were accessed between April and July 2019 to extract variables for this study. Key indicators and sources are outlined in table 1.

**Table 1:** Study indicators and sources

Indicator	Source
Mortality rates	
Maternal mortality rate	UN-MMEIG^53^ UN-IGME^54^ IHME-GBD^55^
Child mortality rate (neonatal, postneonatal, adolescent)	
Coverage level of health interventions	World Bank Development Database,^56^ UN Population Division,^57^ UNICEF Global Database,^58^ WHO^59^
Wealth quintiles	International Centre for Equity in Health^60^
Conflict	
Battle-related deaths	Uppsala Conflict Data Program^5^
Population and social determinants: socioeconomics, maternal education, inequality	International Centre for Equity in Health,^60^ UN Population Division^57^

UN-IGME, United Nations Inter-agency Group for Child Mortality Estimation; UN-MMEIG, United Nations Inter-agency for Maternal Mortality Estimation; IHME-GBD, Institute for Health Metrics and Evaluation – Global Burden of Disease Study.

### Statistical analysis

The number of neonatal and postneonatal deaths and live births were collected for each LMIC for all years between 1990 and 2017, with maternal deaths collected for all years 1990–2015. Annual mortality rates, pooled by conflict status, were generated by summing the deaths across all countries in either conflict group and dividing by the total number of live births. Inequalities in intervention coverage were calculated for three dimensions: wealth, maternal education and place of residence (urban/rural; ‘rurality’). Wealth was estimated from household assets using principal component analysis and organised into quintiles. Maternal education was defined as none, primary and secondary+. Household residence was defined as urban or rural. We examined both absolute and relative metrics of inequality (see box 1^14–16^). For both wealth and maternal education, weighted least squares regression was used to calculate (absolute) Slope Index of inequality (SII) by regressing each indicator’s coverage against the cumulative population distribution midpoint in each group, where weights were proportional to the population size in each group.^17^ Relative inequality measured using Concentration Index was calculated using its standardised formula.^18^ For rurality, absolute inequality was calculated as the difference in intervention coverage between rural and urban populations, and relative inequality was calculated as the ratio of coverage in urban versus rural populations. Country population-weighted absolute and relative inequalities for each intervention were estimated for conflict and non-conflict countries; the most recent estimate for the period 2010–2017 was used. Reproductive and maternal indicators were weighted by the total women of reproductive age, while newborn and child health indicators were weighted with the total under 5 years child population. Linear regression was used to test statistical differences between population-weighted means. P values<0.05 were considered statistically significant. All analyses were conducted using Stata V.14.0.^19^

Box 1 **Defining absolute and relative inequalities**

The concept of inequity refers to the degree of injustice in societies which often results from pervasive inequalities in health or access to services. While inequities are difficult to enumerate and measure, inequalities are easily measurable by examining differences between population subgroups. We examine the objectively measurable inequalities in this study. Inequalities can be expressed in both absolute and relative terms. Relative indicators provide insight into the ratio of unfairness between extreme opposite groups, while absolute indicators measure the actual gap that exists between groups. Both are important for revealing the full picture of inequality^14^ especially for monitoring trends over time since they may yield different trends. For example, a 2018 assessment of reproductive and maternal healthcare services in armed conflict and forced displacement settings in Colombia found that while absolute healthcare inequalities reduced over time, relative inequalities remained unchanged or worsened.^15^

Absolute inequality measures (eg, Slope Index of inequality or SII) ranges from −100% to +100% and denotes the absolute difference in coverage between the extremes of the wealth or education distribution (eg, most educated or richest and least educated or poorest, respectively), with values greater than 0 meaning the top group has greater coverage than the lowest group. Meanwhile, relative inequality (eg, Concentration Index) ranges from −100 to +100, where a value of 0 would represent perfect equality and a positive value would suggest those in the top group have greater relative coverage than the lowest group (with the opposite true of negative values).^16^

Example: Country A is a chronic conflict setting while Country B is a more secure, non-conflict setting. In Country A: if an Individual 1 has a 40% chance of attending an antenatal care (ANC) visit during pregnancy while an Individual 2 has a 75% chance of attending, this would mean the *absolute inequality* between the two is 35% as this is the *absolute* difference between them. Individual 2 is also around twice as likely to attend ANC than Individual 1, and this would be a *relative inequality*. If in Country B: if the same Individual 1 has 40% chance of attending an ANC visit while Individual 2 has 45%, this means *absolute inequality* is only 5%; and there is minimal to no *relative inequality*. This also implies that Country A (conflict country) has greater absolute and relative inequalities than Country B (non-conflict setting).

### Patient and public involvement

Patients were not involved.

## Results

**Figure 2:**
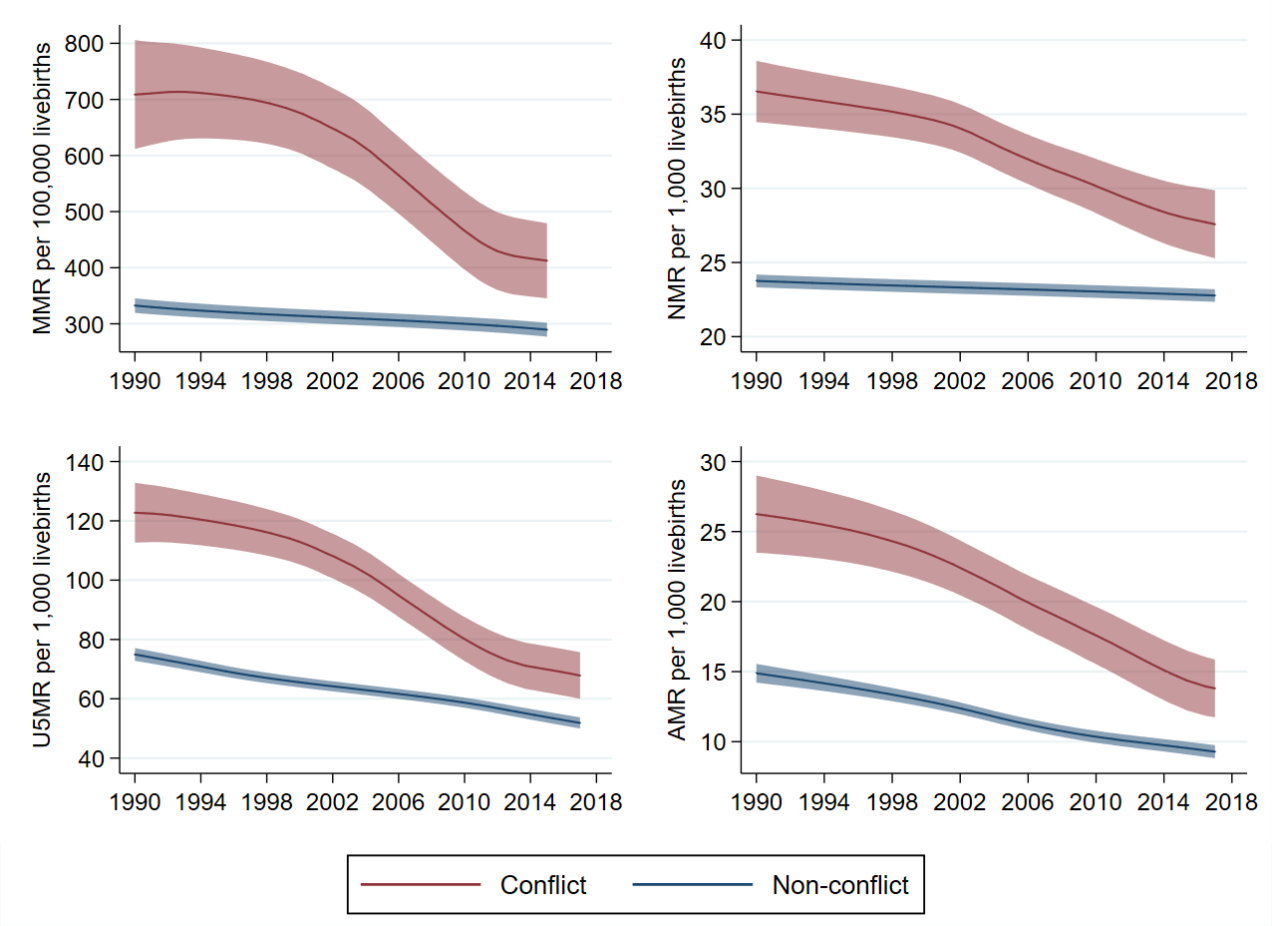
Maternal, newborn, child and adolescent mortality rates for conflict countries (1990–2017) note: UN-based estimates are used for maternal, newborn and child rates; Institute for Health Metrics and Evaluation – Global Burden of Disease Study (IHME-GBD) is used for school-aged children and adolescents (5–14 years). Shaded area indicates 95% uncertainty interval. U5MR, under 5 years mortality rate.

Maternal, newborn (<1 month), young children (1–59 months), and school-aged children and adolescent (5–14 years) mortality rates for the last 30 years have consistently been higher in LMICs experiencing conflict as compared with those with no conflict (figure 2). Five countries, including Afghanistan, Somalia, Sudan, Iraq and Palestine have experienced chronic conflict since 1990 (ie, having BRD rate >10 per million population in each decade since 1990).

We found that in conflict countries, pro-rich and pro-urban inequalities in under 5 years mortality rates were much greater than in non-conflict countries (online supplementary figures A1, A2); in other words, the most disadvantaged children (poorest, rural households) suffer even further in conflict countries. Trends were similar for both absolute and relative measures of inequality (for concept definition see box 1^14–16^). Data for analysis by maternal education were not available for comparison.

**Figure 3:**
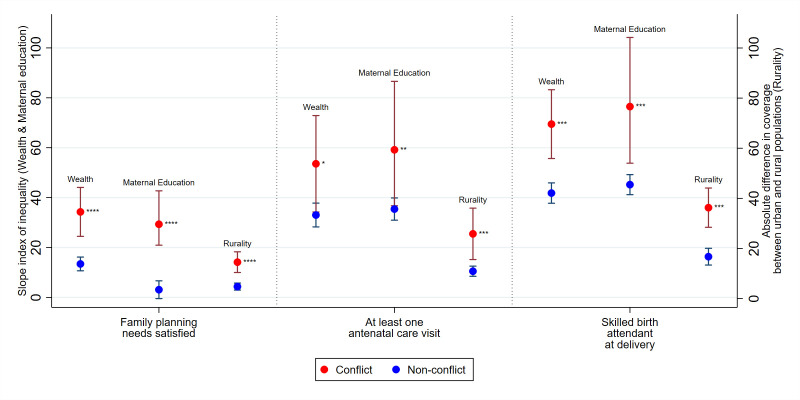
Absolute inequalities in maternal and reproductive health interventions in conflict and non-conflict countries with 95% CIs, 2010–2017, *p<0.05, **p<0.01, ***p<0.001, ****p<0.0001. Note: p values show comparison of mean SII in conflict versus non-conflict countries. Note: larger, positive SII value signals greater pro-rich inequalities between poorest and richest (‘wealth’) or pro-education inequalities between least and most educated (‘maternal education’). Larger, positive absolute difference signals greater pro-urban inequalities between urban and rural mothers (‘rurality’). SII, slope index of inequality.

Inequalities disadvantaging the poorest (vs richest), least educated (vs most educated) and rural mothers (vs urban) are several folds greater in conflict countries compared with non-conflict countries in access to essential reproductive and maternal health services (figure 3, online supplementary table A5). Mean wealth SII for coverage of family planning services was 13 (95% CI 10.7 to 16.2) vs 34 (95% CI 24.5 to 44.1) in non-conflict compared with conflict countries, respectively, representing nearly threefold higher absolute pro-rich inequalities in countries with conflict. Similarly, pro-rich, pro-educated and pro-urban inequalities for ANC during pregnancy and having skilled attendants at delivery (SBA) are also significantly higher in conflict compared with non-conflict countries. Average SII gaps (by wealth, education and rurality) between conflict and non-conflict countries are greatest for SBA (figure 3). Relative inequalities followed similar patterns (online supplementary figure A3). All values for absolute inequalities by wealth, maternal education and rurality can be found in online supplementary table A5.

**Figure 4:**
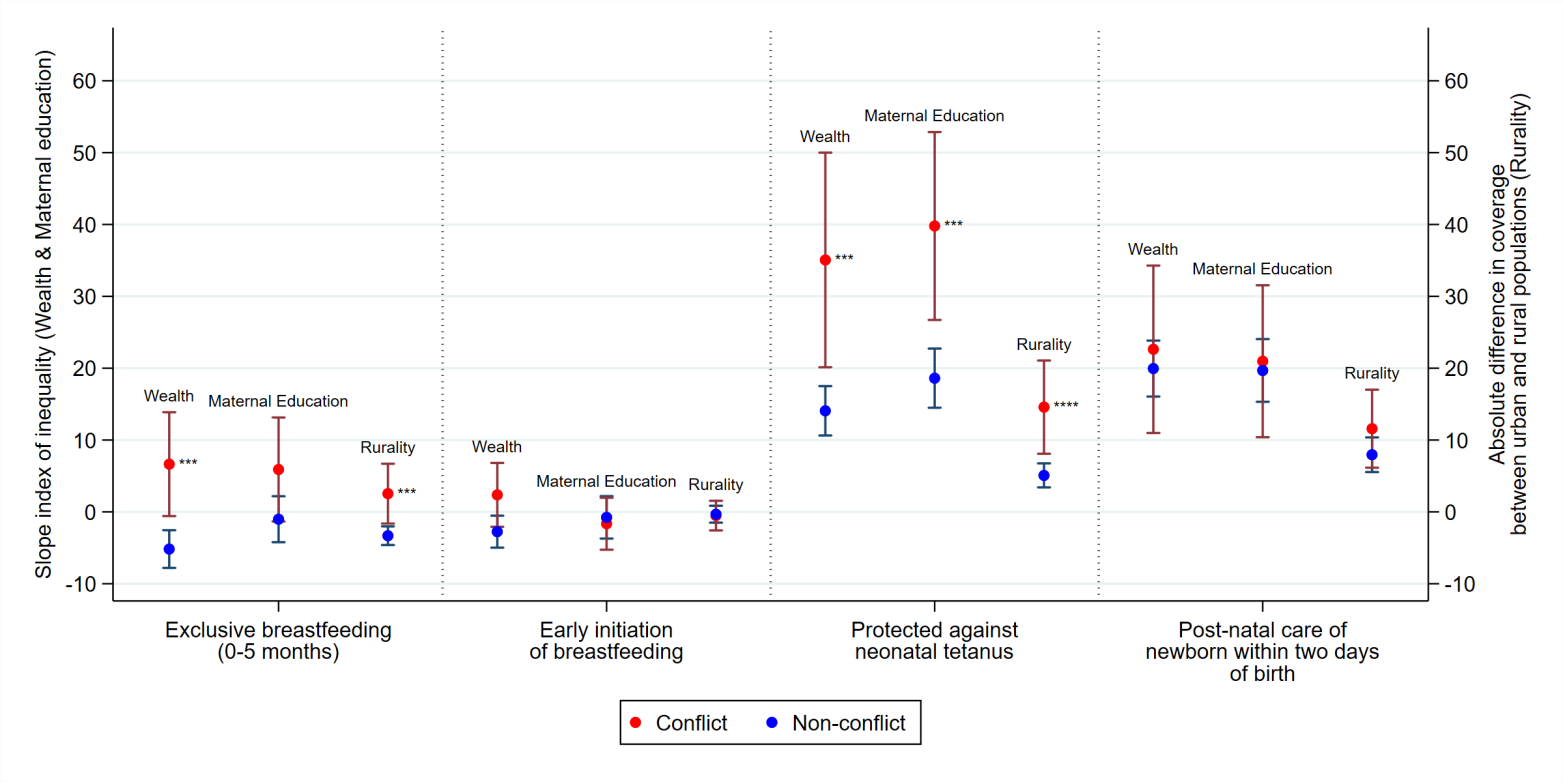
Absolute inequalities in breast feeding and newborn health in conflict and non-conflict countries with 95% CIs, 2010–2017, *p<0.05, **p<0.01, ***p<0.001, ****p<0.0001. Note: p values show comparison of mean SII in conflict versus non-conflict countries. Note: larger, positive SII value signals greater pro-rich inequalities between poorest and richest (‘wealth’) or pro-education inequalities between least and most educated (‘maternal education’). Larger, positive absolute difference signals greater pro-urban inequalities between urban and rural mothers (‘rurality’). SII, slope index of inequality.

With respect to absolute inequalities, the richest and urban populations in conflict countries have better exclusive breastfeeding practices, while in non-conflict countries, poorer and rural households have better coverage (shown by the negative SII values; figure 4). SII in wealth for exclusive breast feeding in non-conflict countries was −5 (95% CI −7.8 to 2.5), while for conflict countries was 6 (95% CI −0.6 to 13.9). The negative value represents pro-poor inequality where those in the lower wealth quintiles have higher levels of exclusive breast feeding, with the opposite being true of positive values. An analogous finding can be observed in inequalities by maternal education with the SII for exclusive breast feeding at −1 (95% CI −4.2 to 2.2) for non-conflict countries, and at 6 (95% CI −1.33 to 13.1) for conflict countries, though this difference was not statistically significant. Relative inequalities follow similar patterns, and more educated mothers in conflict countries are also found to have higher exclusive breast feeding (online supplementary figure A4). Interestingly, inequalities in early initiation of breast feeding follow a different pattern, with generally no differences between conflict and non-conflict countries observed by wealth, maternal education or rurality (figure 4). In terms of relative inequalities, there was a slight difference observed by wealth where conflict countries had pro-rich inequalities and non-conflict countries had pro-poor inequalities (online supplementary figure A4).

Absolute inequalities in neonatal tetanus coverage were significantly more pro-rich, pro-educated mothers and pro-urban in conflict countries (figure 4), and relative inequalities followed similar patterns (online supplementary figure A4). SII for neonatal tetanus coverage in wealth for conflict countries was 35 (95% CI 20.1 to 50.0), and for non-conflict countries was 14 (95% CI 10.6 to 17.5), indicating that while both have pro-rich inequalities, non-conflict countries’ inequalities are greater. Inequalities by maternal education and rurality were akin to those by wealth.

Pro-rich, pro-educated and pro-urban absolute inequalities exist for immediate postnatal care of newborns for both conflict and non-conflict countries (SII~20) with minimal differences across conflict status (figure 4). This suggests newborn babies from advantaged households were more likely to receive postnatal care in general, irrespective of conflict. However, given the general low coverage of postnatal care in LMICs, relative inequalities were notably higher in conflict compared with non-conflict countries, though in the same direction (online supplementary figure A4).

**Figure 5:**
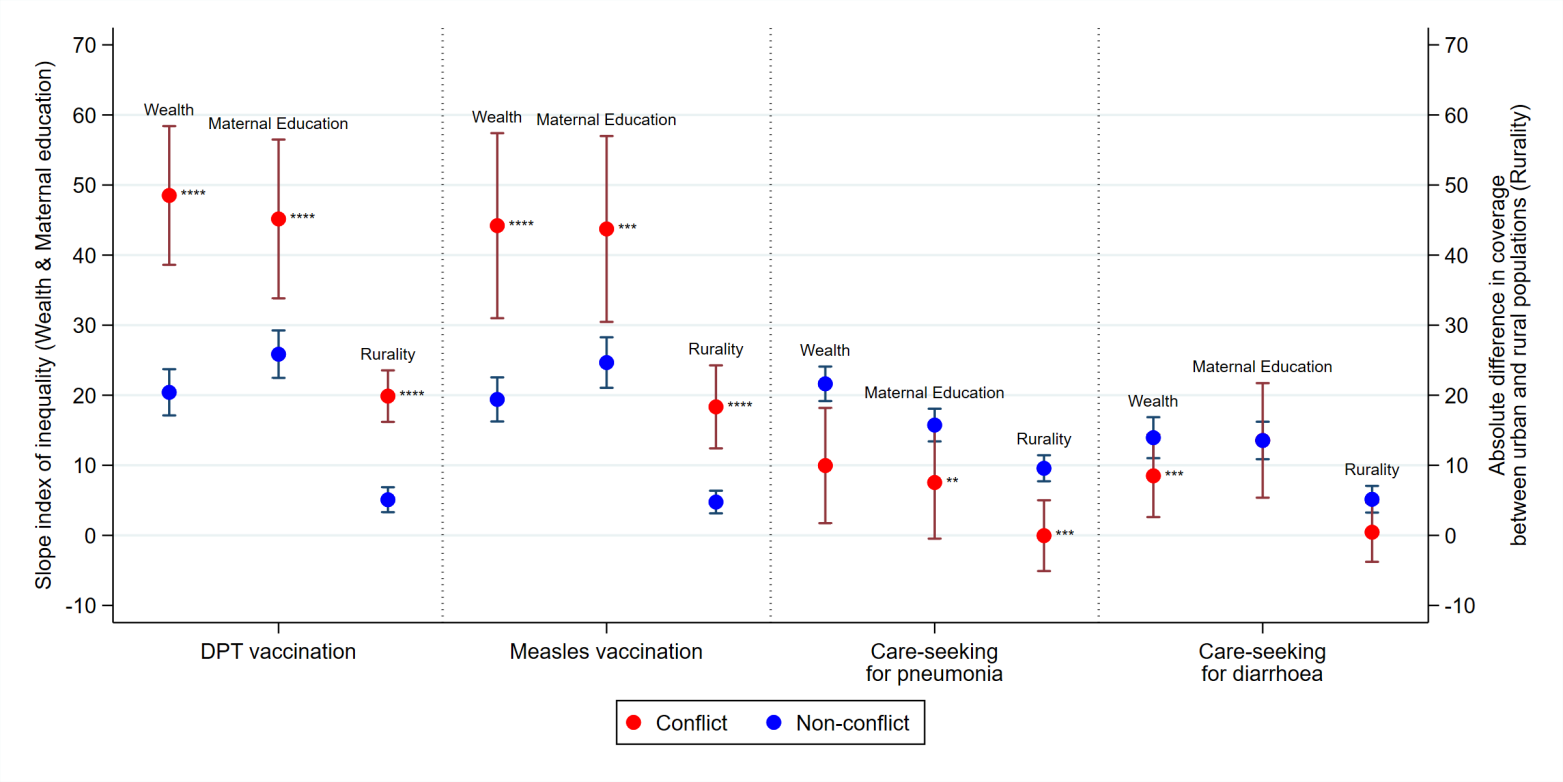
Absolute inequalities in preventative and curative measures of childhood diseases in conflict and non-conflict countries with 95% CIs, 2010–2017, *p<0.05, **p<0.01, ***p<0.001, ****p<0.0001. Note: p values show comparison of mean SII in conflict versus non-conflict countries. Note: larger, positive SII value signals greater pro-rich inequalities between poorest and richest (‘wealth’) or pro-education inequalities between least and most educated (‘maternal education’). Larger, positive absolute difference signals greater pro-urban inequalities between urban and rural mothers (‘rurality’). DPT, diphtheria, pertussis and tetanus vaccine; SII, slope index of inequality.

We found that pro-rich, pro-education and pro-urban inequalities in access to measles and DPT3 vaccination were far greater in conflict countries when compared with non-conflict countries (figure 5, online supplementary figure A5). SII in wealth for DPT vaccination in conflict countries was 49 (95% CI 38.6 to 58.4), compared with 20 (95% CI 17.1 to 23.7) in non-conflict countries. Similarly, for maternal education, SII for DPT vaccination in conflict countries was 45 (95% CI 33.8 to 56.5) compared with 26 (95% CI 22.5 to 29.2) for non-conflict countries. Inequalities by rurality for child vaccinations were low in non-conflict countries but higher in conflict countries.

Strikingly, when it came to careseeking behaviour for pneumonia (ie, whether healthcare was sought for children with symptoms of suspected pneumonia), children from the poorest, least educated and rural households were more likely to seek care in conflict relative to non-conflict countries, while inequalities in careseeking for childhood diarrhoea were not meaningfully different by conflict status (figure 5, online supplementary figure A5).

## Discussion

To our knowledge, this is the first systematic assessment of inequalities in maternal, newborn and child mortality and health service coverage between conflict and non-conflict countries across a prolonged period. We found that mortality and service coverage inequalities (particularly for reproductive and maternal health and child vaccinations) were disproportionately higher in conflict countries by wealth, maternal education and rural residence.

Poverty, illiteracy, pervasive unemployment and unstable governance are often concurrently rampant in these countries, further contributing to and compounding the cycle of violence. Not surprisingly, the most vulnerable are left to fend for survival in environments with higher risks of disease, disability and death.^2^ In sub-Saharan Africa, under 5 years mortality rates in conflict settings have been found to be on average 19 deaths per 1000 live births higher than the regional mean.^20^ While we found that adolescent mortality rates are demonstrably higher in many conflict countries, figures may underestimate the true cost of conflict as there may be under-reporting of those abducted, tortured and forced to become child soldiers, especially common in conflicts in sub-Saharan Africa and parts of South-East Asia.^21^ Negative consequences of conflict on child and maternal mortality is not limited to the active period and immediate location of combat. A recent analysis in African countries found that armed conflict was associated with between 3.0% and 26.7% increased risk of child death within 1 year, with risk extending up to 100 km away from the area of combat and lasting up to 8 years postconflict.^22^

During active conflict, both the supply and demand side of health services are affected. Lower utilisation of health services in conflict areas may be related to poor transportation access, knowledge on where to seek care, low women’s empowerment, insecurity and financial constraints.^23^ Civilians who are financially or socially advantaged have the opportunity to seek required services and to migrate safely.^24^ At a population level, geographical disparities in access to healthcare, gender and ethnicity-based inequities, large-scale population displacement, poor health financing support mechanisms, and reduced capacity and political will for equitable health policy making emerge in conflict settings.^6 25 26^ The increasing urbanisation of civil wars further perpetuates instability country-wide and hampers migration options for families fleeing war. In an analysis of the UCDP data set, Höglund *et al* found that violent conflict occurs more often in urban areas compared with rural ones, and suggested that this was linked to higher population densities and the economic and symbolic relevance of urban spaces.^27^ We can observe this trend in Uganda, where violent political revolts had been commonplace among rural-based rebel groups but, since 2000, urban riots have increased most notably^28^ across Africa as urban populations respond to lack of capacity and poor governance resulting in ‘fragile cities’.^29^ Beyond the direct impact of conflict, literature suggests that pathways to inequalities are compounded and operate through contextual factors; specifically, state governance (measured through government effectiveness, rule of law, control of corruption and regulatory quality) has been linked to health inequalities given its close interplay with peace and stability.^30^

Our findings for health service interventions are evident in country experiences. During periods of widespread conflict in both Nepal and Iraq, women with less education and from lower socioeconomic standing were less likely to access an SBA at delivery,^24^ with similar results found in Colombia.^15^ Øtsby *et al* found that both geographical and temporal proximity to armed conflict significantly decreased the mother’s likelihood of delivering in a healthcare facility, with the greatest risk among poorer, less-educated mothers.^10^

In Nepal, greater incidents of conflict were strongly associated with a lower number of ANC visits during pregnancy.^31^ Additionally, fewer ANC visits during pregnancy were found to be strongly associated with delays in seeking emergency obstetric and newborn care in Afghanistan.^24^ This delay, combined with increased prevalence of both high-risk abortions and prenatal infections, and poorer maternal nutrition during times of conflict,^21^ could result in more maternal and newborn deaths. Arguably, a lack of information on maternal and newborn health service usage in conflict areas means it may be difficult to truly assess the quality of ANC received.^23^

Factors that influence the observed inequalities in exclusive breast feeding may be the inappropriate provision/distribution of breast milk substitutes as part of humanitarian aid, lack of lactation support to mothers, and the stress associated with inability of mothers to exclusively breast feed during conflict. Literature suggests that breastfeeding practices in LMICs are better in poorer and rural households,^32 33^ and our findings supported this in non-conflict countries. The opposite was true for conflict countries; this is in line with a study on breast feeding in violent conflict situations in Iraq which found that breastfeeding incidence reduced as casualties from armed conflict rose.^34^ This finding could be explained by the reduced health staff,^11 25 35^ user fees,^6 25 36^ and geographical barriers^25^ arising from conflict, as many women are counselled in breastfeeding practices,^34 37^ and support from health professionals has been found to be a key determinant of breast feeding.^34^ Alternately, mothers in conflict countries who have been displaced could have impaired nutritional intake, thus reducing breastmilk quality and a decline in breast feeding. Also, conflict leads to male war-related casualties, which can force women into the labour force, thus reducing their time and ability to breast feed.^34^ This would be particularly relevant for women of lower socioeconomic status.

Given the dependence of postpartum healthcare visits on a functioning health system, exacerbated relative inequalities could result from shortages in health staff, financial and geographical barriers, as well as overall deterioration of the health system.^11 25 35 36^ In Syria, maternal and neonatal health service inequalities were greatest by maternal education, rurality and wealth, with the regions under the control of ISIS experiencing greatest access challenges and inequalities favouring advantaged families.^9^

Tetanus immunisation coverage has been found to decrease during conflict.^25^ In west Darfur, protection against neonatal tetanus was shown to improve with an NGO’s enhancement of maternal, newborn and child health programme; however, improvements were not uniform, as interventions did not reach those living in remote communities, or in areas experiencing continued armed conflict or a lack of security.^38^ Another study in Sudan found that child vaccination coverage correlated with unequal household asset distributions, with conflict states having worse outcomes than non-conflict states.^39^

Our study also found that the most disadvantaged populations had lower child vaccination coverage in conflict countries compared with their counterparts in non-conflict countries. These findings are perhaps not too surprising since, due to the methods of vaccine distribution in LMICs, vaccination indicators generally show lower level of inequalities than other indicators. Conflict-affected populations are at particularly high risk for vaccine-preventable disease outbreaks given poor health infrastructure that can compromise delivery of routine immunisation services and make treatment far less accessible.^40^ Further compounding this, the resulting overcrowding and unsanitary conditions, for example, from large-scale population displacements and informal settlements, encourage the spread of disease. Indeed, polio remains endemic in conflict-affected areas with political instability and transient populations such as in Afghanistan and Pakistan.^41^ The 2011 civil war in Syria heavily impacted the country’s previously very high vaccination coverage, particularly in areas most affected by conflict and younger children were at highest risk of incomplete immunisation.^42^ It has been reported that DPT vaccination declined from a preconflict coverage level of 80% in 2010 to 41% in 2015, and there has also been resurgence of polio cases among Syrian refugees entering Turkey.^43^

Unsanitary conditions combined with increased malnutrition can lead to a higher burden of other illnesses such as diarrhoea and pneumonia, which are leading causes of death among young children in humanitarian emergencies,^44^ as well as further challenges with infection prevention/control and subsequent complications including the emergence of antimicrobial resistance and sepsis. Our findings regarding inequalities in careseeking for diarrhoea and pneumonia are only indicative of healthcare behaviours for common childhood diseases as the survey recall questions are only crude measures of treatment of sick children. Current evidence suggests that irrespective of ongoing conflict, families are likely to seek care for these easily treatable conditions. Possible reasons include the nature of care required which typically necessitates one-time visits (ie, repeat visits/dose less likely), as well as these basic treatments often being available in communities, particularly in conflict zones where humanitarian agencies assist with protecting basic health services (especially curative care) and provide essential commodities.^44 45^ Additional research is required to further explore underlying mechanisms for these outcomes.

### What can be done?

Strategies to address disparities and reach marginalised populations in conflict settings will need to use all possible options available to response agencies and public sector programmes.^6 25 36^ Non-state actors typically provide temporary health services in conflict zones, and evidence from several settings substantiates their critical role in bridging health gaps.^24 46–48^ In Afghanistan, for instance, NGOs have been central to health service provision since 2003, and in most provinces (31 out of 34), they are the sole health providers.^46^ Despite continued insecurity and conflict nationwide, maternal and child survival and access to health services has improved considerably in Afghanistan for 16 years, partly due to the success of NGOs in providing services and the availability of high levels of donor funding.^49^ Harnessing the availability, funding and unique social networks of such NGOs provides a valuable opportunity for governments and donors to ensure health equity in the wake of conflict.

Additionally, using community-based approaches including mobile outreach, community health workers and mass-delivered interventions are undoubtedly essential to reaching the hard to reach.^50^ In many settings, with shortages of trained health workers, expansion of this community health workforce, by adapting to or allowing for illiterate/innumerate workers to partake in healthcare may be necessary. An example of expanding the modes of healthcare delivery in conflict settings is the increased use of telemedicine in Syria.^51^ The training of emergency relief workers is also crucial, as they need to be able to conduct rapid needs assessments, establish public health programme priorities, work closely with communities and train local workers, while functioning effectively in hostile and dangerous environments.^52^ Delivery of health services to heard-to-reach conflict-affected people can be delivered through negotiated ceasefires, which have been used in other conflict settings to successfully vaccinate against polio.^52^ The range of mass-delivered interventions can also be expanded to include presumptive antimalarial treatment, mass measles and polio vaccination, tetanus vaccination of pregnant women, treatment of diarrhoeal diseases and treatment of malnutrition.^52^ In other instances, as conditions stabilise and health services are rebuilt, additional preventive and health promotion interventions can be introduced in both non-camp and camp areas. It is also critically important to understand and resolve the root causes of conflict at both the micro (individual, family, community) and macro (structural) levels to customise sustainable preventative and remediating solutions to conflict.^36^ Addressing health inequities in the long term necessitates addressing state fragility since peace and effective governance underpin the development of equitable health systems.^6^

## Limitations and future research

We have only scratched the surface of this complex topic, additional research is required to answer deeper questions, especially at the subnational or regional level in fragile countries. For example, the extent to which conflict aggravates previously existing inequalities in reproductive, maternal, newborn, child, and adolescent health and nutrition (RMNCAH+N) and, consequently, how different stakeholders’ sustained focus is best spent. This is likely to be a common phenomenon as the ability of households to mitigate impact of conflict is likely to be closely linked to socioeconomic status. The extent to which conflicts have an impact on RMNCAH+N inequalities has only been measured in a small number of specific studies which are very informative, but more detailed country-specific studies are needed. Our multicountry analysis based on a classification of countries based on 10-year rates of BRDs undoubtedly leads to some misclassification as conflicts may switch on and off, and conflict duration and severity is not captured. Data availability and quality is also a serious challenge, especially during the active conflict phase. We also did not examine the impact of urbanisation of conflicts or conduct subnational analyses, even though chronic conflicts are often very local and shifting. Rural populations were most disadvantaged as shown by our analyses, but the urban poor are another population requiring further research. We used an ecological approach (ie, at country level) thereby limiting causal or individual-level inferences. We are unable to quantify the impact of conflicts on the magnitude of inequalities in mortality or coverage in our study, as is a challenge for many other studies that do not have a, often impractical, longitudinal design. Our multicountry analyses showed that inequalities in essential health interventions were greater in conflict compared with non-conflict countries for many critical indicators, which presents an additional challenge for the response aiming to reach all with effective interventions, and justifies targeting of the most disadvantaged populations in conflict zones. Additionally, though we have grouped conflict and non-conflict countries to highlight absolute gaps between such settings, it should be underscored that often the structural and contextual make-up (eg, governance, education) across conflict countries varies notably. Such differences should be considered when designing effective interventions and responses to address inequalities in each country.

## Conclusion

This study provided an initial systematic exploration of inequalities in maternal and child health services in conflict settings. Our study has found that for wealth, maternal education and rurality, inequalities tend to be greater in conflict countries compared with non-conflict countries for key RMNCH+N indicators. Our results support efforts to protect reproductive and maternal health and child vaccine interventions in conflict settings, particularly for the most disadvantaged families. These findings should be considered when designing effective contingencies to ensure long-term health protection plans in acute or chronic conflict settings.
