# Supplementary material for: Women, children and adolescents in conflict countries: an assessment of inequalities in intervention coverage and survival
Source: BMJ Glob Health. 2020 Jan 26;5(1):e002214. doi: 10.1136/bmjgh-2019-002214 (PMC7042600; doi:10.1136/bmjgh-2019-002214)
Supplement: Supplementary data [file bmjgh-2019-002214supp001.pdf]

Web Appendix

Table A1: Example conflict classification for Afghanistan and Sudan. Cells highlighted in green show individual years in which the country would be classified as non-conflict while cells in red show years in which they would be classified as conflict. Overall conflict status for each decade is given at bottom of table

Battle-related death rate per 1,000,000 population

Year	Sudan	Afghanistan						
1990	215.2	120.7	2000	104.3	260.5	2010	29.4	244.0
1991	63.0	254.1	2001	122.0	177.7	2011	39.9	249.9
1992	49.9	305.8	2002	70.5	1.4	2012	37.7	251.8
1993	41.8	246.5	2003	67.9	28.6	2013	16.1	253.6
1994	14.4	552.6	2004	108.6	29.5	2014	22.7	376.9
1995	41.5	322.6	2005	5.2	64.9	2015	32.7	511.5
1996	92.3	178.3	2006	31.7	182.8	2016	33.2	517.1
1997	146.0	348.0	2007	6.7	257.1	2017	3.2	532.1
1998	188.1	331.6	2008	18.1	207.2			
1999	52.9	238.6	2009	10.5	227.9			
**Decade average**	**90.5**	**289.9**	**Decade average**	**54.5**	**143.8**	**Decade average**	**26.9**	**367.1**
**Conflict status**	**Conflict**	**Conflict**	**Conflict status**	**Conflict**	**Conflict**	**Conflict status**	**Conflict**	**Conflict**

1

Table A2: Conflict countries for period 2010-2017 based on mean battle-related death rate across decade of at least 10.0 per million individuals

Note: Countries in **bold** suffered severe conflict (i.e. BRD rate of >40 deaths per million population), else moderate (i.e. BRD rate of 10-40 deaths per million population)

	Period 2010-2017
Country	Battle-related death rate per 1,000,000 individuals
**Afghanistan**	**367.1**
Central African Republic	12.2
**Iraq**	**190.4**
**Libya**	**92.2**
Mali	13.6
Nigeria	11.5
Pakistan	13.5
**Somalia**	**130.4**
**South Sudan**	**49.9**
Sudan	26.9
**Syria**	**1757.0**
Turkey	10.7
Ukraine	17.5
**Yemen**	**82.6**

Akseer N, *et al. BMJ Global Health* 2020; 5:e002214. doi: 10.1136/bmjgh-2019-002214

Table A3: Number of countries classified as conflict and non-conflict for each year 1990-2017 based on annual battle-related death rate of at least 10 per million individuals

Year	Non-conflict	Conflict	Year	Non-conflict	Conflict
1990	115	20	2004	126	9
1991	113	22	2005	129	6
1992	113	22	2006	125	10
1993	118	17	2007	129	6
1994	116	19	2008	124	11
1995	119	16	2009	126	9
1996	119	16	2010	128	7
1997	119	16	2011	124	11
1998	115	20	2012	121	14
1999	115	20	2013	124	11
2000	115	20	2014	120	15
2001	119	16	2015	117	18
2002	123	12	2016	121	14
2003	124	11	2017	122	13

Table A4: Number of countries classified as conflict and non-conflict for each decade 1990-2017 based on mean battle-related death rate of at least 10 per million individuals per decade

Decade	Non-conflict	Conflict
1990-1999	100	35
2000-2009	117	18
2010-2017	121	14

Figure A1: Relative inequalities in under-5 mortality rates in conflict and non-conflict countries by wealth (left) and rurality (right), 2010-2018
* p < 0.05, ** p < 0.01, *** p < 0.001, **** p < 0.0001

Note: Since under-5 mortality is an adverse outcome, a greater negative number indicates more inequality favouring the poorest or rural populations (i.e. these groups experience higher death rates); rurality is presented on a separate axis (right) measuring relative inequalities using the ratio of coverage between urban and rural estimates.

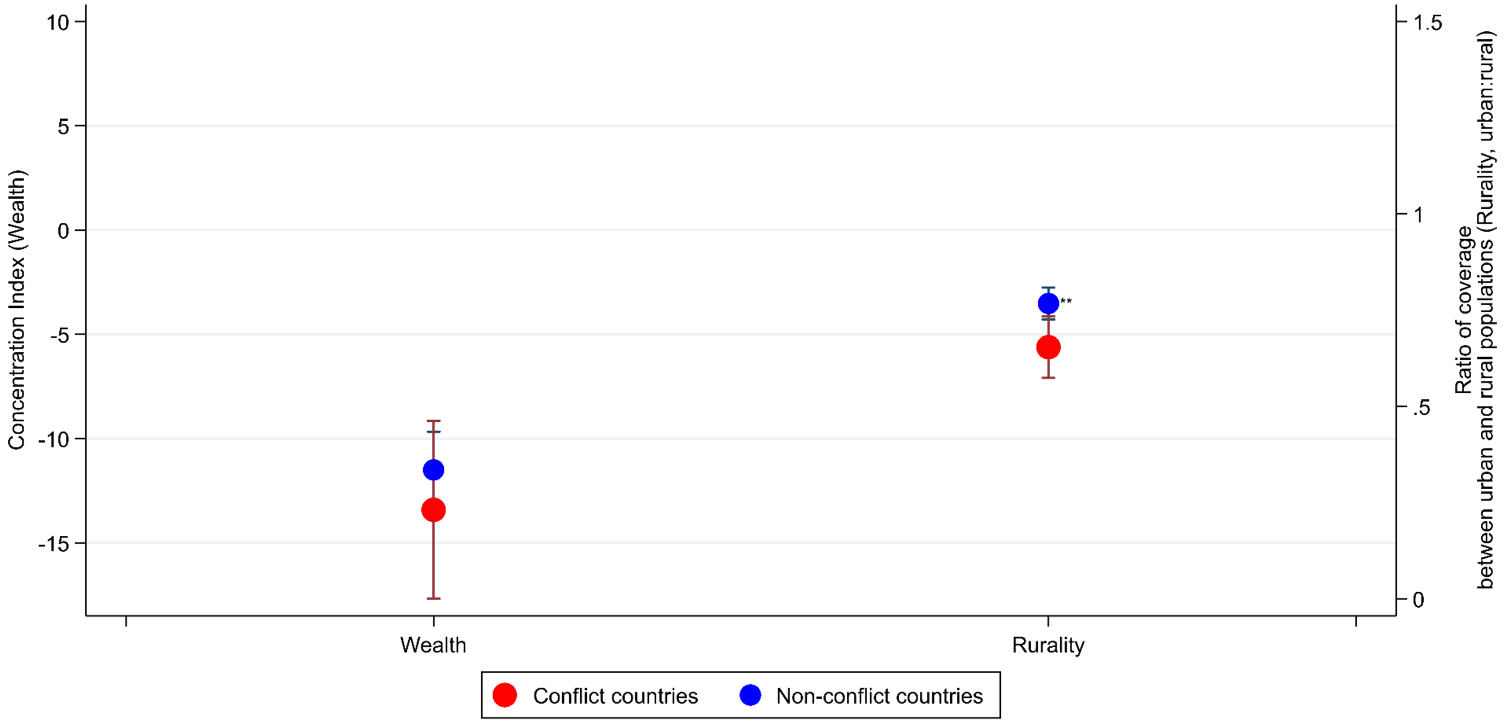

Akseer N, *et al. BMJ Global Health* 2020; 5:e002214. doi: 10.1136/bmjgh-2019-002214

Figure A2: Absolute inequalities in under-5 mortality rates in conflict and non-conflict countries by wealth (left) and rurality (right), 2010-2018
* p < 0.05, ** p < 0.01, *** p < 0.001, **** p < 0.0001

Note: Since under-5 mortality is an adverse outcome, a greater negative number indicates more inequality favouring the poorest or rural populations (i.e. these groups experience higher death rates); rurality is presented on a separate axis (right) measuring absolute inequalities via gaps in coverage; under-5 mortality data by maternal education was not available.

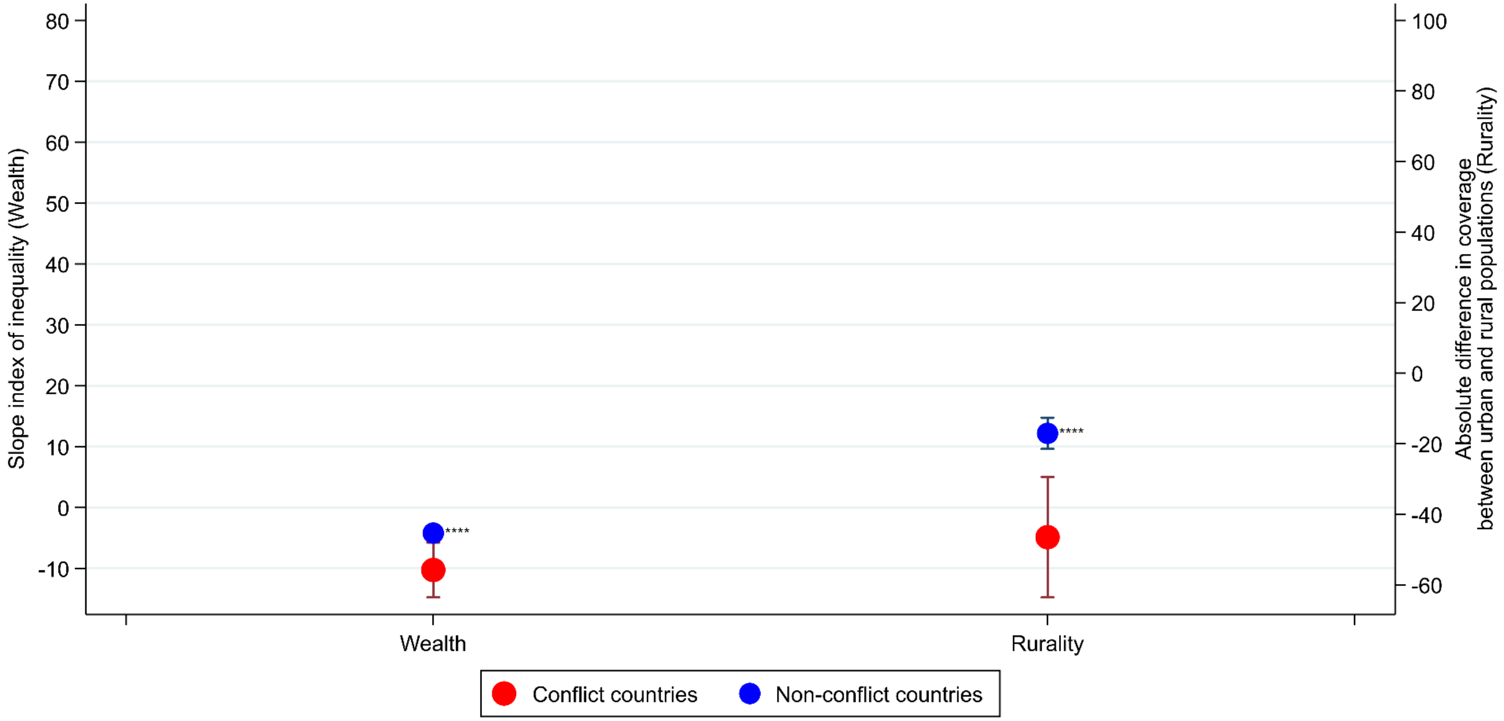

Akseer N, *et al. BMJ Global Health* 2020; 5:e002214. doi: 10.1136/bmjgh-2019-002214

Table A5: Absolute inequalities in wealth, maternal education and rurality for select maternal, newborn and child health indicators for conflict and non-conflict countries

	Family Planning Needs Satisfied	At least 1 antenatal care visit	Skilled birth attendant at delivery	Exclusive breastfeeding (0-5 months)	Early initiation of breastfeeding	Protected against neonatal tetanus	Postnatal care of newborn within 2 days of birth	DPT vaccination	Measles vaccination	Careseeking for pneumonia	Careseeking for diarrhea
**Wealth Inequalities**											
Mean in non-conflict	13.42	33.02	41.82	-5.18	-2.76	12.33	10.43	20.41	19.40	21.63	13.94
Mean in conflict	34.27	53.55	69.45	6.64	2.37	33.69	28.39	48.51	44.20	9.96	8.50
Overall mean	16.03	35.25	44.81	-2.93	-1.81	14.87	13.47	25.61	23.99	19.46	13.03
Non-conflict country n	78	51	51	48	80	66	79	76	76	75	65
Conflict country n	12	7	7	7	13	12	13	13	13	13	9
p-value	<0.0001	0.0107	0.0001	0.0004	0.0541	0.0001	<0.0001	<0.0001	<0.0001	0.0005	0.1344
**Maternal Education Inequalities**											
Mean in non-conflict	3.09	35.41	45.20	-1.02	-0.76	16.30	19.68	25.86	24.66	15.74	13.54
Mean in conflict	29.32	59.16	76.49	5.90	-1.65	38.67	20.97	45.15	43.73	7.54	13.55
Overall mean	6.43	38.04	48.66	0.31	-0.93	19.00	19.90	29.48	28.24	14.19	13.54
Non-conflict country n	75	49	49	76	78	64	46	74	74	71	64
Conflict country n	12	7	7	13	13	12	7	13	13	13	9
p-value	<0.0001	0.0029	0.0001	0.0704	0.7896	0.0004	0.8143	<0.0001	0.0002	0.0091	0.9983

	Family Planning Needs Satisfied	At least 1 antenatal care visit	Skilled birth attendant at delivery	Exclusive breastfeeding (0-5 months)	Early initiation of breastfeeding	Protected against neonatal tetanus	Postnatal care of newborn within 2 days of birth	DPT vaccination	Measles vaccination	Careseeking for pneumonia	Careseeking for diarrhea
**Rurality Inequalities**											
Mean in non-conflict	4.79	10.92	16.73	-3.32	-0.32	3.99	7.96	5.08	4.76	9.58	5.15
Mean in conflict	14.54	25.83	36.28	2.54	-0.51	14.17	11.58	19.87	18.34	-0.04	0.45
Overall mean	6.00	12.53	18.85	-2.21	-0.36	5.19	8.57	7.82	7.27	7.78	4.36
Non-conflict country n	78	51	51	79	81	67	48	77	77	69	65
Conflict country n	12	7	7	13	13	12	7	13	13	13	9
p-value	p<0.0001	0.0001	0.0003	0.0006	0.8868	p<0.0001	0.2319	p<0.0001	p<0.0001	0.0001	0.0505

Akseer N, *et al. BMJ Global Health* 2020; 5:e002214. doi: 10.1136/bmjgh-2019-002214

Figure A3: Relative inequalities in reproductive and maternal health interventions in conflict and non-conflict countries, 2010-2018
* p < 0.05, ** p < 0.01, *** p < 0.001, **** p < 0.0001
Note: p-values show comparison of mean CIX / ratio (for rurality) in conflict vs. non-conflict countries

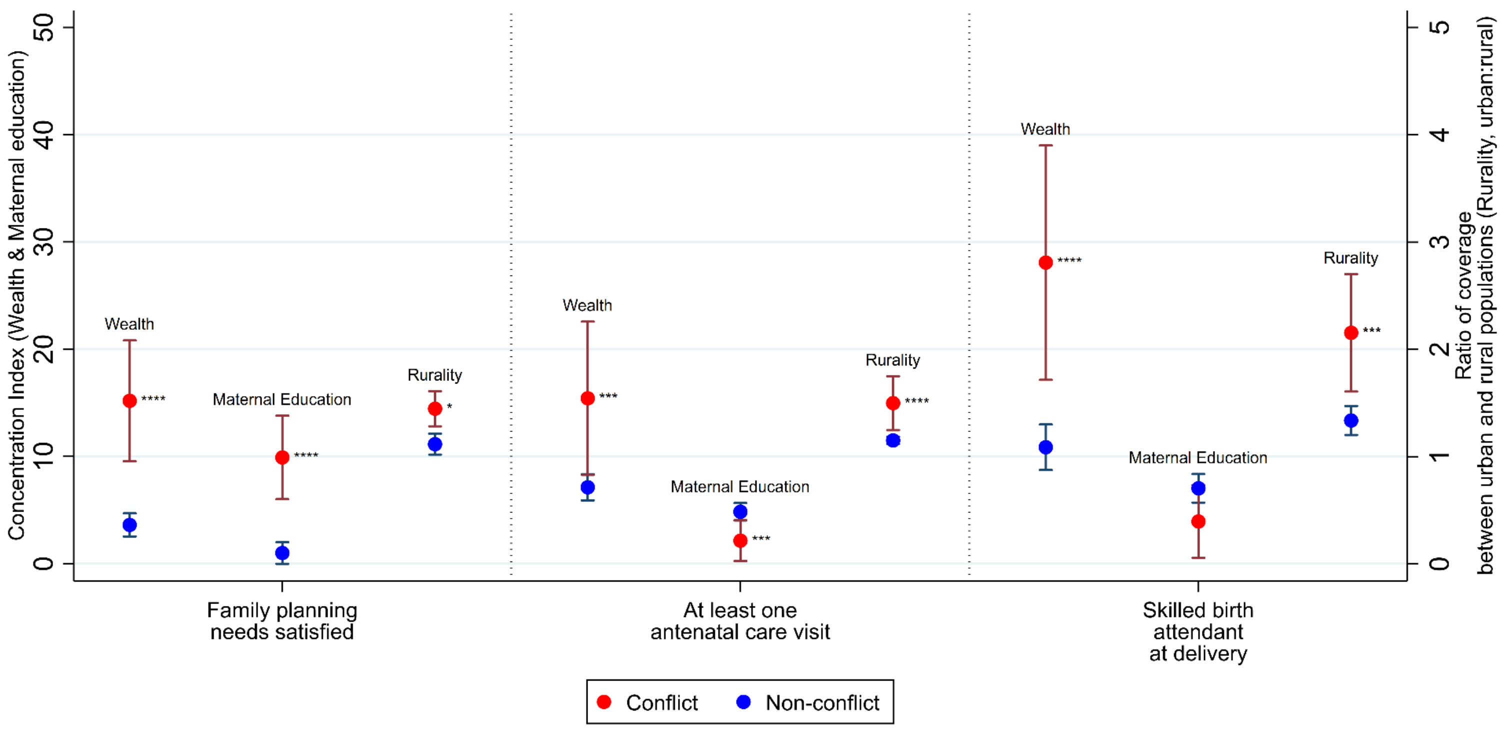

Note: Larger, positive SII value signals greater inequalities between poorest and richest ("Wealth") or least and most educated ("Maternal Education"). Larger, positive ratio signals greater inequalities between urban and rural mothers ("Rurality")

Figure A4: Relative inequalities in breastfeeding and newborn health in conflict and non-conflict countries, 2010-2018
* p < 0.05, ** p < 0.01, *** p < 0.001, **** p < 0.0001
Note: p-values show comparison of mean CIX / ratio (for rurality) in conflict vs. non-conflict countries

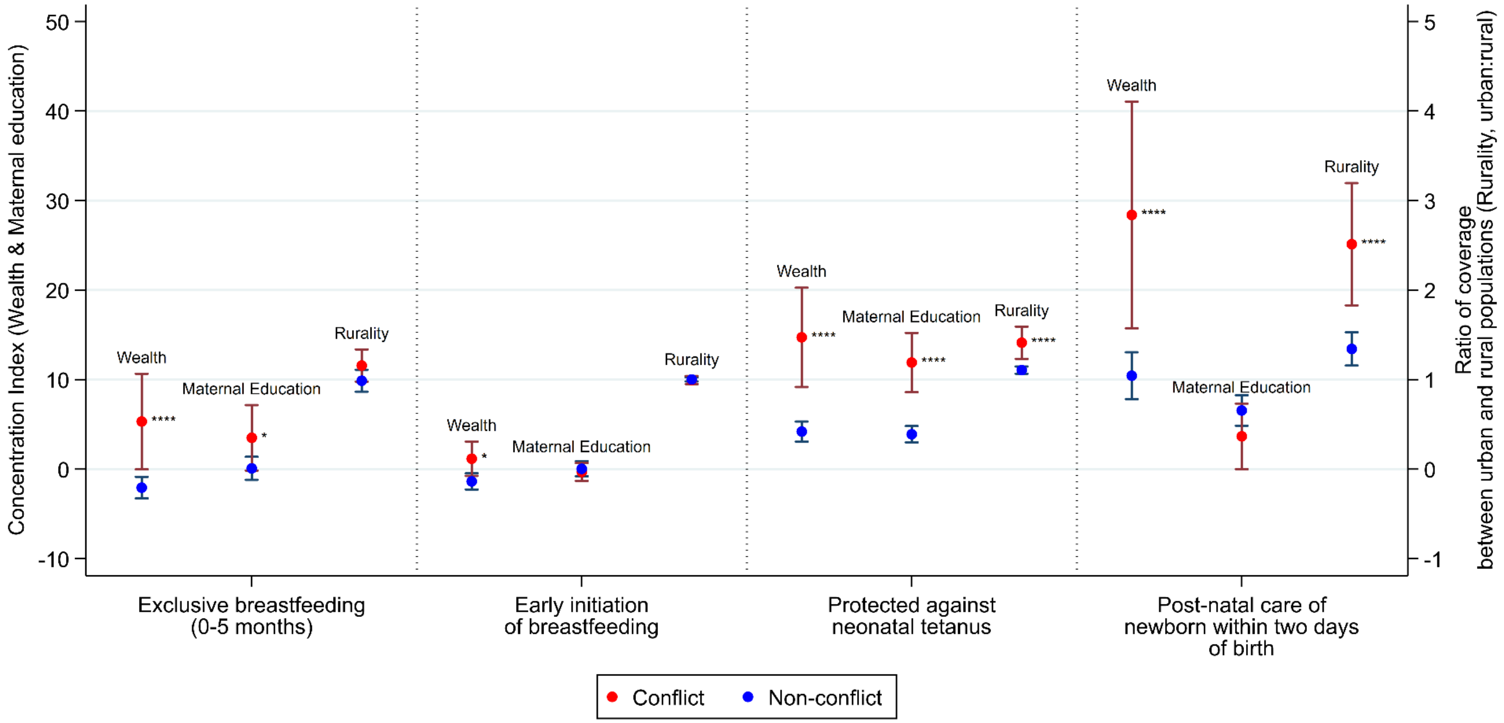

Note: Larger, positive SII value signals greater inequalities between poorest and richest ("Wealth") or least and most educated ("Maternal Education"). Larger, positive ratio signals greater inequalities between urban and rural mothers ("Rurality")

Figure A5: Relative inequalities in preventative and curative measures of childhood diseases in conflict and non-conflict countries, 2010-2018
* $p < 0.05$, ** $p < 0.01$, *** $p < 0.001$, **** $p < 0.0001$
Note: p-values show comparison of mean CIX / ratio (for rurality) in conflict vs. non-conflict countries

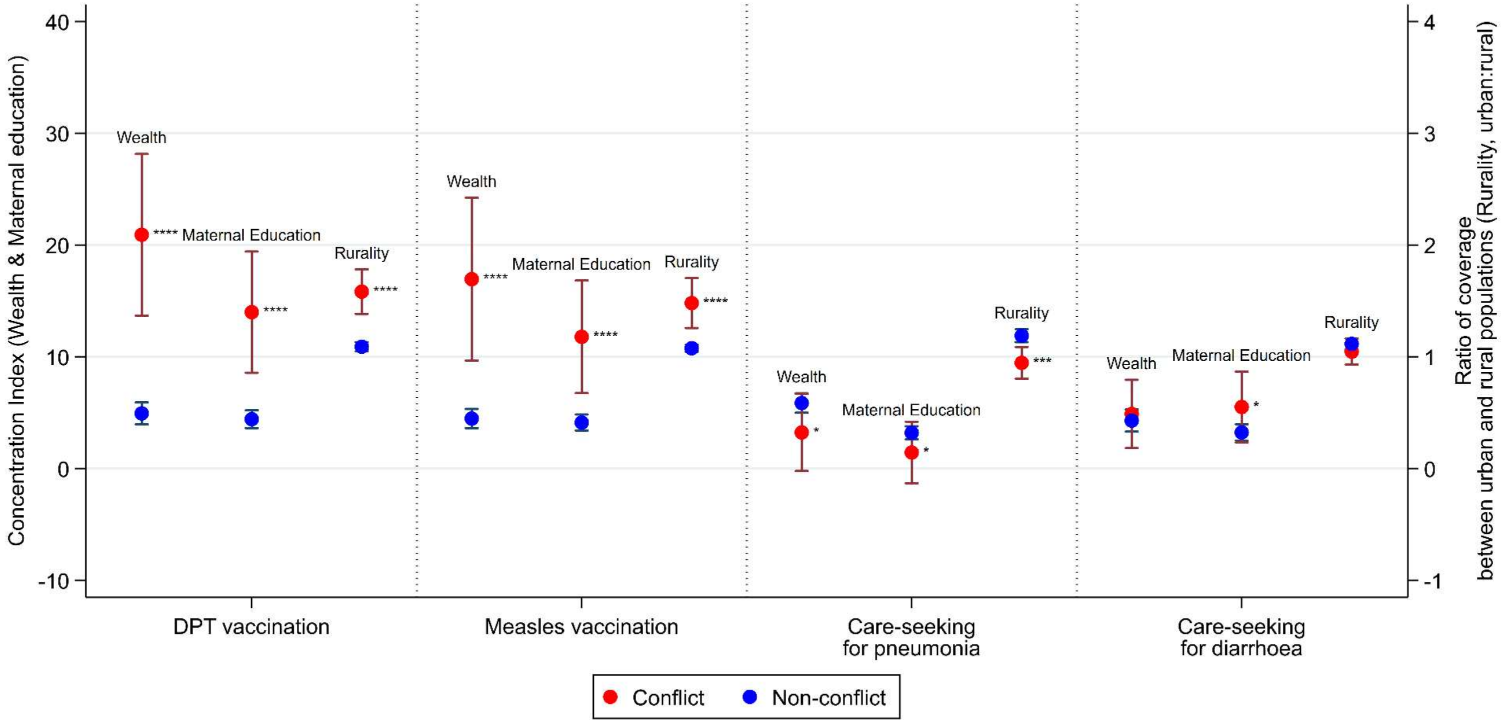

Note: Larger, positive SII value signals greater inequalities between poorest and richest ("Wealth") or least and most educated ("Maternal Education"). Larger, positive ratio signals greater inequalities between urban and rural mothers ("Rurality")

Akseer N, *et al. BMJ Global Health* 2020; 5:e002214. doi: 10.1136/bmjgh-2019-002214
